# Computing hemodynamic response functions from concurrent spectral fiber-photometry and fMRI data

**DOI:** 10.1117/1.NPh.9.3.032205

**Published:** 2022-01-05

**Authors:** Tzu-Hao H. Chao, Wei-Ting Zhang, Li-Ming Hsu, Domenic H. Cerri, Tzu-Wen Wang, Yen-Yu I. Shih

**Affiliations:** a University of North Carolina at Chapel Hill, Center for Animal MRI, Chapel Hill. North Carolina, United States; b University of North Carolina at Chapel Hill, Biomedical Research Imaging Center, Chapel Hill. North Carolina, United States; c University of North Carolina at Chapel Hill, Department of Neurology, Chapel Hill. North Carolina, United States; d University of North Carolina at Chapel Hill, Department of Biomedical Engineering, Chapel Hill. North Carolina, United States

**Keywords:** Hemodynamic response function, fMRI, Fiber-photometry, rat, multi-modal, MRI compatible

## Abstract

**Significance:**

Although emerging evidence suggests that the hemodynamic response function (HRF) can vary by brain region and species, a single, canonical, human-based HRF is widely used in animal studies. Therefore, the development of flexible, accessible, brain-region specific HRF calculation approaches is paramount as hemodynamic animal studies become increasingly popular.

**Aim:**

To establish an fMRI-compatible, spectral, fiber-photometry platform for HRF calculation and validation in any rat brain region.

**Approach:**

We used our platform to simultaneously measure (a) neuronal activity via genetically encoded calcium indicators (GCaMP6f), (b) local cerebral blood volume (CBV) from intravenous Rhodamine B dye, and (c) whole brain CBV via fMRI with the Feraheme contrast agent. Empirical HRFs were calculated with GCaMP6f and Rhodamine B recordings from rat brain regions during resting-state and task-based paradigms.

**Results:**

We calculated empirical HRFs for the rat primary somatosensory, anterior cingulate, prelimbic, retrosplenial, and anterior insular cortical areas. Each HRF was faster and narrower than the canonical HRF and no significant difference was observed between these cortical regions. When used in general linear model analyses of corresponding fMRI data, the empirical HRFs showed better detection performance than the canonical HRF.

**Conclusions:**

Our findings demonstrate the viability and utility of fiber-photometry-based HRF calculations. This platform is readily scalable to multiple simultaneous recording sites, and adaptable to study transfer functions between stimulation events, neuronal activity, neurotransmitter release, and hemodynamic responses.

## 1 Introduction

Our interpretation of functional MRI data is built on the assumption that neuronal and vascular responses are tightly coupled.^1^^,^^2^ The process by which changes in local neural activity lead to changes in cerebral blood flow (CBF) and blood volume^3^ is often termed neurovascular coupling and this relationship can be described mathematically with a hemodynamic response function (HRF). Early fMRI studies typically assigned a single canonical human HRF across brain regions (hereafter as canonical HRF), derived from two gamma functions.^4^ However, emerging evidence shows that neurovascular coupling can be substantially different between species, brain regions, and physiological conditions.^5^^–^^10^ Thus, use of the canonical HRF could lead to inaccurate interpretation of fMRI data, especially in preclinical studies that often utilize anesthetized non-human species. Some recent fMRI studies have adapted to use empirically derived HRFs (hereafter as empirical HRFs), but these current approaches have notable limitations (to be discussed in the next paragraph), and a majority of studies still rely on the canonical HRF.^11^^–^^13^ Therefore, the development of techniques to acquire flexible, accessible, brain-region specific HRF is paramount as the number of hemodynamic animal studies continues to grow.

To date, there are two distinct approaches commonly used to obtain empirical HRFs. (1) multi-modal measurements, wherein neuronal and vascular activity are measured simultaneously and the HRF is obtained by deconvolution of the two signals;^14^ and (2) MRI-data driven approaches, which recreate representative hemodynamic responses from repeated observations of hemodynamic changes relative to the timings of external stimuli, spontaneous events, or state changes.^15^^–^^19^ While MRI-data driven approaches can be applied to most datasets, because they rely on computational modeling rather than ground-truth data, HRF estimates will differ according to the choice of model (linear or non-linear) and associated parameters, which are often the subject of debate. Conversely, the multi-modal measurement approach most often combines fMRI with electrophysiology for simultaneous measurement of hemodynamics and ground-truth neuronal activity.^20^^–^^25^ One major challenge of this approach is that electrophysiology signal is extremely sensitive to Eddy current-induced noise from fMRI acquisitions, making it very difficult to get acceptable quality signals.^20^^–^^25^ Further, while electrophysiology tools provide high temporal resolution (up to kHz) for measuring neuronal activity, this resolution is seldom beneficial to HRF calculations because the sampling rate of fMRI hemodynamic information is typically on the order of seconds.

Emerging optical approaches like multi-photon microscopy and wide-field optical imaging have become popular for multimodal measurement of neuronal activity^26^^–^^34^ and hemodynamic signals^26^^–^^28^ without the need for fMRI. These approaches have high sampling rate (∼10 to 40 Hz^26^^–^^33^) and provide spatial distribution information, making them suitable for measuring multiple brain regions simultaneously.^27^^,^^28^ However, signal loss through brain tissue greatly limits the detection depth of both multi-photon microscopy^26^^,^^29^^–^^33^ and wide-field optical imaging.^27^^,^^28^ For example, wide-field imaging and two-photon microscopy can commonly reach a detection depth of just 400 to 500  μm from the brain surface,^26^^–^^28^^,^^31^ while three-photon microscopy can reach deeper to ∼1200  μm,^29^^,^^30^^,^^32^^,^^33^ slightly beyond cortical depth in mice. Many subcortical brain regions (e.g., striatum, thalamus, amygdala, etc.) are still beyond reach.

An alternative optical measurement approach is fiber-photometry, which makes use of the same optical fiber to excite fluorescent proteins and receive emitted photons from targeted brain areas. In an obvious advantage over other optical techniques, fiber-photometry can be used anywhere inside the brain with minimal tissue damage along the fiber tract (typically 100 to 250  μm in diameter). Further, this technique is readily MRI-compatible, and with the recent advent of genetically encoded calcium indicators such as GCaMP6f, it is relatively simple to obtain genetically defined neuronal activity during fMRI,^35^^–^^41^ offering an excellent opportunity to shed light on neurovascular coupling mechanisms in both cortical and subcortical structures. However, multimodal recording of neuronal and hemodynamic activity with only fiber-photometry, circumventing the temporal limitations and complexity of fMRI, has not been well-established. To further advance the field, we established a multi-channel, spectral fiber-photometry platform, which allows simultaneous measurement of multiple fluorescent sources (e.g. GCaMP6f and a red-shifted fluorescent vascular dye), in multiple brain regions.^42^^,^^43^ Importantly, this platform enables measurement of the full emission spectrum with improved accuracy, which is crucial for the quantification of spectrally overlapped fluorescent signal changes.

In the current study, we multiplexed our multi-channel, spectral fiber-photometry platform with fMRI to measure three sources of neurophysiological signal at the same time: (a) neuronal activity from genetically encoded calcium indicators (GCaMP6f) expressed on principal neurons, (b) photometry cerebral blood volume (photometry-CBV) from intravenously administered red fluorescent dye (Rhodamine B), and (c) fMRI cerebral blood volume (fMRI-CBV) from fMRI with a vascular contrast agent (Feraheme). First, we benchmark the use of Rhodamine B and evaluated the signal changes and contrast-to-noise ratio (CNR) of photometry-CBV and fMRI-CBV within the primary somatosensory cortex (S1) during forepaw stimulation. Next, we computed an empirical HRF for S1 from spontaneous neuronal activity and photometry-CBV changes in the absence of stimulation, and determined the optimal data length for HRF calculation, demonstrating the ability of our platform to calculate empirical HRFs from resting-state photometry data. Then we cross-validated our findings by direct comparison of empirical HRFs derived from simultaneously acquired photometry-CBV and fMRI-CBV signals aligned to forepaw stimulation. Having validated our photometry-derived HRF method, we assessed the utility of this platform over the use of the canonical HRF within a general linear model (GLM) to detect significant whole-brain activity changes to forepaw stimulation in fMRI data. Finally, we used our platform to calculate HRFs from resting-state data from several other brain areas, including the prelimbic cortex located deeper in the brain, and show the performance of these empirical HRFs versus the canonical HRF in GLM detection of brain-wide networks from fMRI data.

## 2 Materials and Methods

### 2.1 Subject

**Fig. 1:**
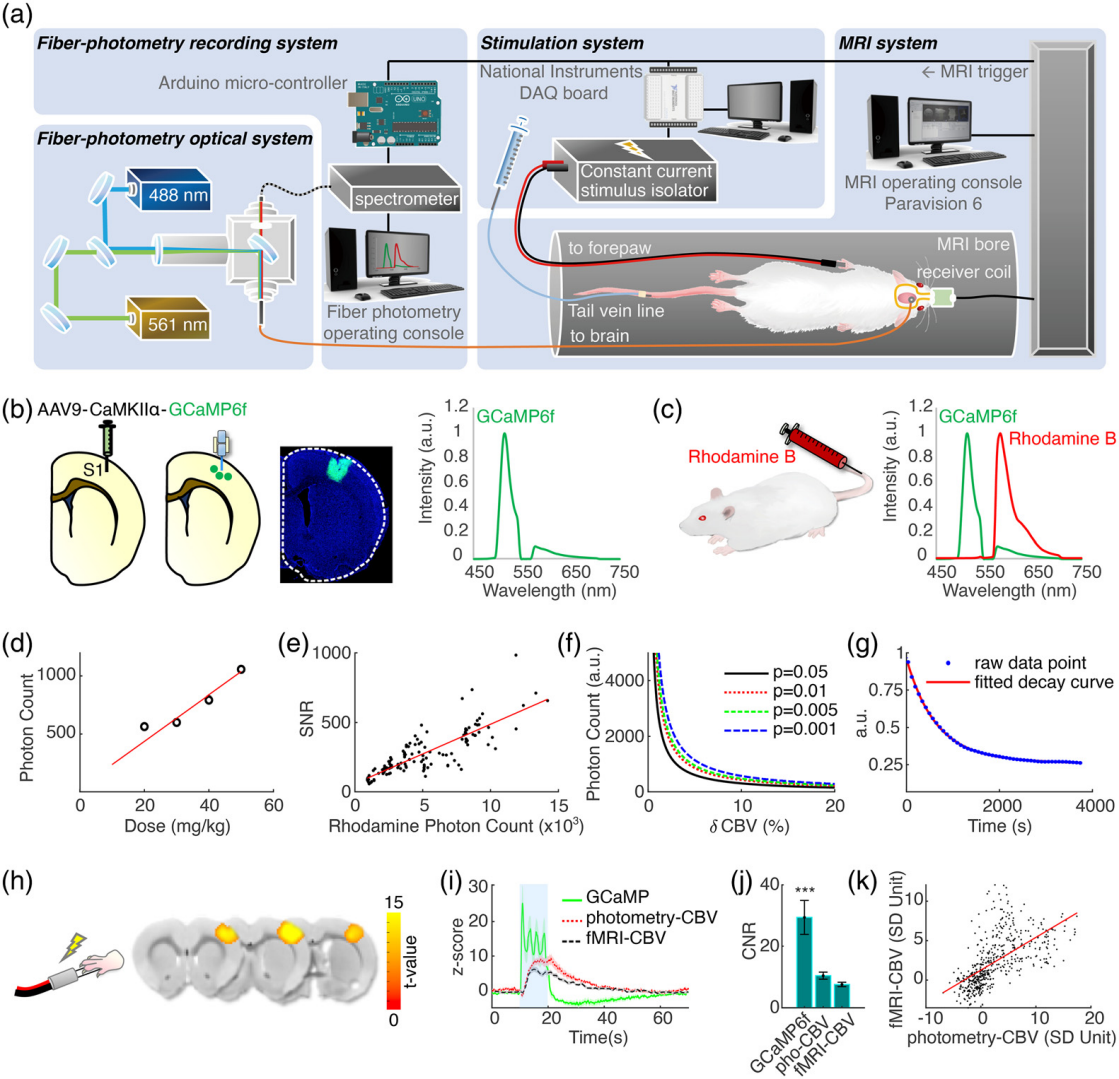
(a) The setup of the spectral fiber-photometry platform synchronized with a 9.4T small animal MRI system. 488- and 561-nm laser light is combined and delivered to the rat brain through a fiber optic cable connected to an implanted optical fiber. Fluorescence emission signal returns through the same cable, then is delivered to a spectrometer for recording, which is synchronized in time to 1 Hz fMRI acquisition by TTL pulses, upsampled to 10 Hz through an Arduino board. The stimulation system for electrical forepaw stimulation is also synchronized to fMRI acquisition and is controlled by a separate PC with a DAQ board. (b) GCaMP6f is expressed via microinjection of genetically engineered AAV into the target brain area. An optical fiber is implanted 0.3 mm above the injection site. The GCaMP6f emission wavelength has a peak at 515 nm. (c) To monitor CBV activity with photometry, Rhodamine B is injected via tail vein catheter. GCaMP6f and Rhodamine B spectra are unmixed to derive their coefficients for quantification. (d) Multi-dose test revealed that Rhodamine B spectral peak photon counts are linearly correlated with Rhodamine B injection dose (mg/kg). (e) The SNR and spectral peak photon counts of Rhodamine B recordings are linearly correlated. (f) Rhodamine B photon counts needed for detecting CBV changes of various magnitudes at different statistical thresholds. (g) The half-life of Rhodamine B clearance following bolus injection is measured at 698.3 s. (h) fMRI-CBV response maps to the electrical forepaw stimulation paradigm consisting of a 60 s initial baseline period followed by two sets of 10 s electrical forepaw stimulation blocks (9 Hz, 2.5 mA, 0.5 ms) with 60 s resting periods after each block. (i) Time-courses of GCaMP6f (green), photometry-CBV (red) and fMRI-CBV (black) from S1, aligned to electrical forepaw stimulation. (j) Photometry-CBV has higher CNR than fMRI-CBV (p<0.01), and GCaMP shows the highest CNR. The CNR is calculated by dividing the evoked response peak value with the standard deviation of baseline fluctuation (GCaMP: 25.36±5.31, Rhodamine-CBV: 7.93±1.4, fMRI-CBV: 6.18±0.74). (k) Photometry-CBV and fMRI-CBV peak response amplitudes to electrical forepaw stimulation are linearly correlated. Demo of simultaneous multimodal fiber-photometry and fMRI recordings in S1 (Video 1, MOV, 8 MB [URL: https://doi.org/10.1117/1.NPh.9.3.032205.1]).

This study employed a total of 9 wild type male Sprague Dawley (SD) rats and 10 Thy1-GCaMP6f transgenic male Long-Evans (LE) rats weighing between 300 and 600 g. All procedures were performed in accordance with the National Institutes of Health Guidelines for Animal Research (Guide for the Care and Use of Laboratory Animals) and approved by the University of North Carolina (UNC) Institutional Animal Care and Use Committee. The SD rats were separated into two cohorts. In the first cohort (n=5), GCaMP was expressed in the right forelimb S1 (S1FL) using AAV9-CaMKIIα-GCaMP6f-WPRE-SV40 ($\text{titer}\geq 1\times 10^{13}\;\mathrm{vg}/\mathrm{mL}$, Penn Vector Core). This cohort of rats underwent multi-dose test of Rhodamine B to optimize the Rhodamine B dose for photometry-CBV recording [Figs. 1(d)–1(g)], and received electrical forepaw stimulation to validate the evoked GCaMP and Rhodamine B signal changes [Figs. 1(h)–1(k)]. In the second cohort (n=4), GCaMP and hM3Dq (a Gq-DREADD) were co-expressed in the right S1FL using a mixture of AAV9-CaMKIIα-GCaMP6f-WPRE-SV40 ($\text{titer}\geq 1\times 10^{13}\;\mathrm{vg}/\mathrm{mL}$, Penn Vector Core) and AAV5-CaMKIIα-hM3Dq-mCherry ($\text{titer}\geq 2\times 10^{12}\;\mathrm{vg}/\mathrm{mL}$, Addgene) at 1:1 ratio to demonstrate that the HRF is state-dependent with and without DREADD. In Fig. 6, we used Thy1-GCaMP6f transgenic rats^47^ (n=10) expressing the fluorescent calcium activity indicator, GCaMP6f,^14^ under the Thy1 promoter,^48^ allowing measurement of cortical output activity from pyramidal neurons. All rats in this study were housed under environmentally controlled conditions (12 h normal light/dark cycles, lights on at 7am; 20°C to 23°C and 40% to 60% relative humidity), with ad libitum access to food and water.

### 2.2 Stereotactic Surgery

All coordinates used in this study are listed as follows. S1: AP=+0.5  mm and ML=+3.7  mm, DV=1.3  mm, prelimbic cortex (PrL): AP=3.3  mm and ML=0.8  mm, DV=3.5  mm, anterior cingulate cortex (ACC): AP=1.5  mm and ML=0.8  mm, DV=2  mm, retrosplenial cortex (RSC): AP=−2.2  mm and ML=0.7  mm, DV=2  mm, anterior insular cortex (AI): AP=3.2  mm and ML=4.2  mm, DV=3.5  mm. For all surgical procedures, rats were anesthetized initially by 5% isoflurane and maintained by a constant flow of 2-3% isoflurane mixed with medical air. Rectal temperature was continuously monitored and maintained within 37°C±0.5°C using a feedback-controlled heating pad (Harvard Apparatus, Model 557020, Holliston, MA). For the first two cohorts of experiments, SD rats were head-fixed to a stereotactic frame (Kopf Instruments, Model 962, Tujunga, California). The skin was opened to expose the skull surface, and burr holes were prepared according to experimental coordinates. Microinjections were performed at a flow rate of 0.1  μl/min for 1  μl, and an additional 10 min was given for virus diffusion prior to slow retraction of the microsyringe needle. The burr holes were then sealed with bone wax (Fisher Scientific, Pittsburgh, PA), and the wound was sutured. A month after the virus microinjection, the skin was reopened to expose the skull, then the bone wax was removed and optical fibers (200  μm in diameter; NA: 0.39) were chronically implanted to coordinates 0.3 mm above the virus injection sites. We did not identify any significant size change in adult rat brains during the 1-month virus incubation before fiber implantation according to our recent study,^49^ thus no re-adjustment of the coordinates for fiber implantation was applied. Four MR-compatible miniature brass screws (Item #94070A031, McMaster Carr, Atlanta, Georgia) were anchored to the skull, then the surface of the skull was covered with dental cement to seal implanted components and the wound was sutured to further protect the surgical site. The screws and dental cement helped hold the implanted fibers firmly on the skull. For the third cohort of the experiment that used transgenic LE rats, most of the surgical procedurals were the same as the first two cohorts except no virus was microinjected. At the end of every surgical procedure, lidocaine jelly (#866096, Henry Schein Inc., Melville, New York) was applied around the surgical wound for pain relief and to prevent the rat scratching the wound. Meloxicam (#6451720670, Henry Schein Inc., Melville, New York) was also given by oral administration for further pain relief. Rats were allowed at least one week for recovery from surgical procedures before any further experiments.

### 2.3 Experimental Setup

The spectrally resolved fiber-photometry system in this study replicates an established system described previously.^42^^,^^43^ Laser beams from a 488 nm 60 mW continuous wave (CW) laser (OBIS 488 LS-60, Coherent, Santa Clara, California) and a 561 nm 50 mW CW laser (OBIS 561 LS-50, Coherent, Inc.) are aligned and combined by broadband dielectric mirrors (BB1-E02, Thorlabs, Newton, New Jersey) and a long-pass dichroic mirror (ZT488rdc, Chroma Technology Corp), then launched into a fluorescence cube (DFM1, Thorlabs, Newton, New Jersey). Extra neutral density filters (NEK01, Thorlabs, Newton, New Jersey) are placed between the combined laser beam and the fluorescence cube to adjust the final laser power. The fluorescence cube contains a dichroic mirror (ZT488/561rpc, Chroma Technology Corp) to reflect and launch the combined laser beam through an achromatic fiber port (PAFA-X-4-A, Thorlabs, Newton, New Jersey) into the core of a 105/125  mm core/cladding multi-mode optical fiber patch cable. The distal end of the patch cable is connected to an implantable optical fiber probe for both excitation laser delivery and emission fluorescence collection. The emission fluorescence collected from the fiber travels back along the patch cable into the fluorescence cube, passes through the dichroic mirror and an emission filter (ZET488/561 m, Chroma Technology Corp, Bellows Falls, Vermont), then launches through an aspheric fiber port (PAF-SMA-11-A, Thorlabs, Newton, New Jersey) into the core of an AR-coated 200/230  mm core/cladding multi-mode patch cable (M200L02S-A, Thorlabs, Newton, New Jersey). The AR-coated multi-mode patch cable is connected to a spectrometer (QE Pro-FL, Ocean Optics, Largo, Florida) for spectral data acquisition, which can be operated by a UI software OceanView (Ocean Optics, Largo, Florida). To achieve concurrent recording during fMRI, trigger mode is used in OceanView, where the photometry system is synchronized with MRI using an Arduino micro-controller board. The stimulation system uses a DAQ board (1208Hs-2AO, Measurement Computing Corp., Norton, Massachusetts) to send out stimulus triggers according to the stimulus paradigm set in a homemade software program. During fMRI experiments, the DAQ synchronizes stimulation pulses via triggers from the MRI system. Stimulation pulses were driven by a constant current stimulus isolator (A385RC, World Precision Instruments, Sarasota, Florida) for forepaw electrical stimulation experiments.

### 2.4 Animal Subject Preparation and Physiology Management

General animal subject preparation and maintenance followed the same protocol detailed in our previous publications.^50^^,^^51^ Rats were initially anesthetized with 4% isoflurane (Vaporizer #911103, VetEquip Inc., Livermore, California) mixed with medical air and endotracheally intubated using a 14G x 2“(>400  g) or 16G x 2“(<400  g) i.v. catheter (Surflash Polyurethane Catheter, TERUMO, Somerset, New Jersey). Respiration was maintained by a ventilator (SAR-830 or MRI-1, CWE Inc, Ardmore, PA) set at 60  breaths/min and an inspiration time ratio of 40%. A rectal probe was used to monitor core body temperature (OAKTON Temp9500, Cole-Parmer, Vernon Hills, Illinois) and a capnometer was used to monitor heart rate, peripheral blood oxygen saturation, and end-tidal ${\mathrm{CO}}_{2}$ (SURGIVET^®^ V90041LF, Smith Medical, Dublin, Ohio). Body temperature was maintained at 37°C±0.5°C using a circulating water blanket connected to a temperature adjustable water bath (Haake S13, Thermo Fisher Scientific, Waltham, Massachusetts). Ventilation tidal volume was adjusted to keep the heart rate at 300±50 beats per minute, peripheral blood oxygen saturation above 90%, and end-tidal ${\mathrm{CO}}_{2}$ between 2.8% and 3.2%. End-tidal ${\mathrm{CO}}_{2}$ values from this capnometer system were previously calibrated against invasive sampling of arterial blood gas, reflecting a partial pressure of carbon dioxide (${\mathrm{pCO}}_{2}$) level of 30 to 40 mm Hg.^52^^,^^53^ For studies using Rhodamine B for CBV measurements, a bolus dose of 40  mg/kg (Sigma–Aldrich, St. Louis, Missouri) was injected via tail vein. For DREADD studies, a single dose of clozapine (0.05  mg/kg, Sigma–Aldrich, St. Louis, Missouri) was injected via tail vein.

### 2.5 Concurrent Functional MRI Scan with Fiber-Photometry Recording

All fMRI data in this study were collected on a Bruker BioSpec 9.4-Tesla, 30-cm bore system with 6.0.1 on an AVANCE II console (Bruker BioSpin Corp., Billerica, Massachusetts). An RRI BFG 150/90 gradient insert (Resonance Research, Inc, Billerica, Massachusetts) paired with a Copley C700 gradient amplifier (Copley Controls Corp., Canton, Massachusetts) was used. A homemade single-loop surface coil with an internal diameter of 1.6 cm was used as a radio-frequency transceiver. Isoflurane concentrations were adjusted to 2% and animals were secured in to a custom-built, MR-compatible rat cradle. Animal physiology was monitored and maintained as described in the previous paragraph.

Upon stabilizing the animals, a pair of needle electrodes was inserted under the skin of forepaw for stimulation. Before connecting the fiber-photometry patch cable, all light in the room was turned off, the final output power of 488- and 561-nm laser were adjusted to balance spectral amplitudes.^42^ The maximum power used in this study was <100  μW. Then, a background spectrum was measured as a reference by pointing the fiber tip to a nonreflective background in the dark room. This background spectrum was then automatically subtracted by OceanView during photometry recording. Following setup processes, the cradle was pushed into MRI bore, and a bolus of dexmedetomidine (0.025  mg/kg; Dexdormitor, Orion, Espoo, Finland) cocktailed with paralytic agent rocuronium bromide (4.5  mg/kg; Sigma–Aldrich, St. Louis, Missouri) was injected into the tail vein. Fifteen minutes after the bolus injection, continuous intravenous infusion of dexmedetomidine (0.05  mg/kg/h) and rocuronium bromide (9  mg/kg/h) cocktail was initiated and the isoflurane concentration was adjusted to 0.5% to 1% for the entire scanning period.^54^

Magnetic field homogeneity was optimized first by global shim and followed by local first- and second-order shims according to B0 map. Anatomical images for referencing were acquired using a rapid acquisition with relaxation enhancement (RARE) sequence (12 coronal slices, thickness=1  mm, repetition time (TR)=2500  ms, echo time (TE)=33  ms, matrix size=256×256, field-of-view $(\mathrm{FOV})=25.6\times 25.6\;{\mathrm{mm}}^{2}$, in plane resolution 0.1×0.1  mm, average = 8, RARE factor=8). The center of the 5^th^ slice from the anterior direction was aligned with the anterior commissure. Blood-oxygen-level-dependent (BOLD) fMRI scans were acquired using a multi-slice single-shot gradient echo echo-planar imaging (GE-EPI) sequence (slice  thickness=1  mm, TR=1000  ms, TE=14  ms, matrix size=80×80, $\mathrm{FOV}=25.6\times 25.{6\;\mathrm{mm}}^{2}$, in plane resolution 0.32×0.32  mm, bandwidth=250  kHz). CBV fMRI scans were acquired using a similar GE-EPI sequence with a shorter TE (slice thickness = 1 mm, TR=1000  ms, TE=8.1  ms, matrix size=80×80, $\mathrm{FOV}=25.6\times 25.{6\;\mathrm{mm}}^{2}$, in plane resolution 0.32×0.32  mm, bandwidth=250  kHz). Both fMRI scans were acquired with the same image slice geometry imported from the previously acquired T2-weighted anatomical image. For CBV fMRI, a session of GE-EPI scans with 300 repetitions was taken, and at about the 100^th^ scan, Feraheme (30 mg Fe/kg, i.v.) was administered for CBV percentage change calculations. This data contains the original brain contrast in the first 100 scans before the Feraheme injected, resulting a mean cortical SNR of the averaged first 100 scans as 396.72±30.84. This allows quantification of signal changes caused by the contrast agent and therefore enable calculation of CBV changes (see details in Sec. 2.6). For forepaw stimulation fMRI, a monophasic constant current of 2.5-mA intensity with a 0.5-ms pulse width at a frequency of 9 Hz was applied, and two stimulation paradigms were used in this study: block-design (60 s-off, 10 s-on, 60 s-off, 10 s-on, 60 s-off) and event-related (10 s-off, 1 s-on, 39 s-off, 1 s-on, 39 s-off, 1 s-on, 39 s-off). For resting-fMRI, 600 repetitions (10 min) were scanned as a previous study showed that the reliability of resting-fMRI connectivity estimates reached a plateau in about 9 to 16 min.^55^

At the end of fMRI experiment, the rat was recovered from anesthesia and paralysis by receiving atipamezole hydrochloride (3  mg/kg, i.v.; ANTISEDAN, Orion, Espoo, Finland), for the reversal of the sedative and analgesic effects of dexmedetomidine, and sugammadex sodium (4 to 8  mg/kg, i.v.; Merck Sharp & Dohme Corp., Kenilworth, New Jersey), for the reversal of the paralytic effect of rocuronium.^54^

### 2.6 CBV fMRI Data Processing and Statistical Analyses

All fMRI data were analyzed using the analysis of functional neuroimages (AFNI)^5^ GLM framework.^6^ All EPI images were skull-stripped^56^ and slice-timing was corrected. Then automatic co-registration was applied to realign time-courses data within subjects to correct subtle drift of EPI images. In addition, the resting-state fMRI images were then linearly detrended, high-pass filtered (>0.01  Hz), independent component analysis (ICA) denoise, and head movement regression. The high-pass filter was chosen to retain high frequency power (i.e., >0.1  Hz) and to remove noise generated from respiration and heart rate without significant loss of purported neuronal-based signal.^57^^,^^58^ ICA denoise was used to identify and remove physiological, movement and thermal (machine) noise components.^59^ Finally all EPI images were aligned to a T2-weighted rat brain template^7^ to generate normalized fMRI images to allow for group-level comparisons, and Gaussian smooth (FWHM=0.6  mm) was performed. To test the group-level significant consistency of the stimulus-evoked responses, we employed a parametric one-sample t-test implemented in AFNI. The significant threshold was set to $p_{\text{corrected}}<0.05$ (corrected by 3dClustSim). A region of interest (ROI) of ball with diameter (r=1.2  mm, 16 voxels) was placed at the fiber tip in the S1 to extract fMRI time-course data. To account for Feraheme kinetics over the course of experiments, the following equations were used to calculate the ${\Delta R}_{2}^{*}(baseline)$, ${\Delta R}_{2}^{*}(stim)$, and ΔCBV.^8^

$${\Delta R}_{2}^{*}(baseline)=-\frac{1}{TE}\;\ln(\frac{S_{\text{prestim}}}{S_{0}}),$$ (1)

$${\Delta R}_{2}^{*}(stim)=-\frac{1}{TE}\;\ln(\frac{S_{\text{stim}}}{S_{\text{prestim}}}),$$ (2)

$$\Delta \mathrm{CBV}=\frac{{\Delta R}_{2}^{*}(\text{stim})}{{\Delta R}_{2}^{*}(baseline)},$$ (3)

where $S_{\text{prestim}}$ and $S_{0}$ represents MR signal intensity after and before Feraheme injection, respectively, and $S_{\text{stim}}$ are the MR signal intensities during the stimulation. The brain activation map in Fig. 5 used GCaMP time-course or stimulation paradigm convolved with empirical or canonical HRF as regressor for GLM analysis of fMRI during forepaw stimulation, Fig. 6 used GCaMP time-course convolved with empirical or canonical HRF as regressor for GLM analysis of resting data, and others are the common GLM analysis based on the stimulation paradigm.

### 2.7 Fiber-Photometry Spectral Unmixing

To untangle the GCaMP and Rhodamine spectra, mixed spectra acquired by fiber-photometry were analyzed using a spectral linear unmixing algorithm, which can effectively remove crosstalk between multicolor sensors as shown in previous studies.^42^^,^^43^^,^^49^ Briefly, at any time point n, the mixed spectrum Y(n) was modeled as

$$Y(n)=Coff_{1}(n)\times S_{1}+Coff_{2}(n)\times S_{2}+C+\varepsilon (n),$$ (4)

where $S_{1}$ and $S_{2}$ are the normalized reference emission spectra of the two fluorescence signal sources. ${\mathrm{Coff}}_{1}$ and ${\mathrm{Coff}}_{2}$ are the unknown regression coefficients corresponding to the $S_{1}$ and $S_{2}$ respectively. C is the unknown constant, and ε(n) is random error. $Coff_{1}(n)$, $Coff_{2}(n)$, and ε(n) at each time point were estimated using the lm() function in the RStudio package (RStudio Inc. V1.0.136, Boston, Massachusetts).

### 2.8 Modeling the Hemodynamic Response Function

The relationship between neuronal activity and hemodynamic response can be expressed as:

HbT(T)=N(T)⊗HRF(t)+c+d(T), (5)

where the HbT(T) represents hemoglobin fluctuation time-course, the N(T) represents neuronal activity time-course, the HRF(t) represents an impulse HRF with t sampling points, c is a constant for baseline offset, and d(T) is for linear drift over time. Assuming T={0,1,2,…,m}, t={0,1,2,…,n}, this equation can be expressed as

$$\left[\begin{array}{c} HbT(0)\\ HbT(1)\\ HbT(2)\\ \vdots \\ HbT(m) \end{array}]=[\begin{array}{ccccccc} N(0) & 0 & 0 & & 0 & 1 & 0\\ N(1) & N(0) & 0 & \cdots & 0 & 1 & \frac{1}{m}\\ N(2) & N(1) & N(0) & & 0 & 1 & \frac{2}{m}\\ & \vdots & & \ddots & & \vdots & \\ N(m) & N(m-1) & N(m-2 & \cdots & N(m-n) & 1 & 1 \end{array}]\times [\begin{array}{c} HRF(0)\\ HRF(1)\\ HRF(2)\\ \vdots \\ HRF(n)\\ c\\ D \end{array}\right]$$ (6)

Therefore, the HRF(t), c and D (slope of d(T)) can be solved using Ordinary Least Squares solution with known HbT(T) and N(T). According to our experience, we recommend starting with >1600 sampling points for m and ∼250 sampling points for n when a 10 Hz sampling rate is used. To avoid slow nonlinear drift and physiological noise contamination, HbT(T) were band-pass filtered with cutoff frequencies at 0.01 and 0.5 Hz before the HRF estimation.

### 2.9 Histology

At the end of the experiments, rats were euthanized by a mixture of 1 to 2 ml of sodium pentobarbital and phenytoin sodium (Euthasol, Virbac AH, Inc., Westlake, Texas), and transcardially perfused with saline followed by 10% formalin. The brains were removed and stored in 10% formalin overnight, then transferred into a 30% sucrose solution (in 0.1 M phosphate buffer) for 2 to 3 days, until brains sunk to bottom of storage bottles. These brains were cut into serial coronal sections (40  μm) using a cryotome (#HM450, Thermo Fisher Scientific, Waltham, Massachusetts) and mounted on glass slides. Fluoro-Gel II Mounting Medium (#17985-50, Electron Microscopy Sciences, Hatfield, Pennsylvania) was covered on the brain slides to provide DAPI stain and for fluorescence imaging. Slides were imaged using a Zeiss LSM780 confocal microscope.

### 2.10 Statistical Analysis

All data are expressed as the mean ± standard error. A P value of <0.05 was considered statistically significant. Differences between empirical HRF and canonical HRF were compared by conducting an independent two-sample t test. The CNR among GCaMP6f signal, photometry-CBV and fMRI-CBV were compared through one-way repeated measures ANOVA and Tukey’s post hoc multiple comparison.

## 3 Results

Our concurrent fiber-photometry and fMRI recording platform is shown in Fig. 1(a). We used an AAV vector to express GCaMP6f in S1 under the CaMKIIα promotor for neuronal activity measurement [Fig. 1(b)] and injected a single bolus of Dextran conjugated Rhodamine B (70,000 molecular weight) via a tail vein catheter for CBV measurement [Fig. 1(c)]. The conjugated Dextran group enlarges the molecular size of the Rhodamine B compound for a slower elimination rate from the blood stream and minimizes the baseline drift during CBV recording. Figure 1(c) shows the representative mixed emission spectra of GCaMP6f and Rhodamine B. Mixed spectra time-courses data were recorded at 10 Hz sampling rate, then linearly unmixed offline to derive their coefficients for quantification. Rhodamine B spectral peak signal intensity is dose-dependent [Fig. 1(d)] and is directly related to the signal-to-noise ratio of the photometry-CBV measurement [Fig. 1(e)]. This information allows us to calculate the minimum baseline Rhodamine B signal required to detect CBV changes of various magnitudes [Fig. 1(f)]. We use the spectral peak photon count as the unit shown in Figs. 1(d)–1(f) because it can be easily monitored in real time during the preparation for photometry-CBV recording. The baseline signal decay curve following Rhodamine B bolus injection indicates that roughly 30 min are needed for the signal to reach a steady state [Fig. 1(g)]. Simultaneous multimodal fiber-photometry and fMRI recordings of S1 (Video 1), aligned to electrical forepaw stimulation, showed similar activation patterns of photometry-CBV and fMRI-CBV, concurrent with a robust increase in GCaMP6f neuronal signal, and notably, photometry-CBV had significantly higher CNR than fMRI-CBV [Figs. 1(i)–1(k)]. In addition, fMRI provided brain-wide CBV activation maps [Fig. 1(h)].

**Fig. 2:**
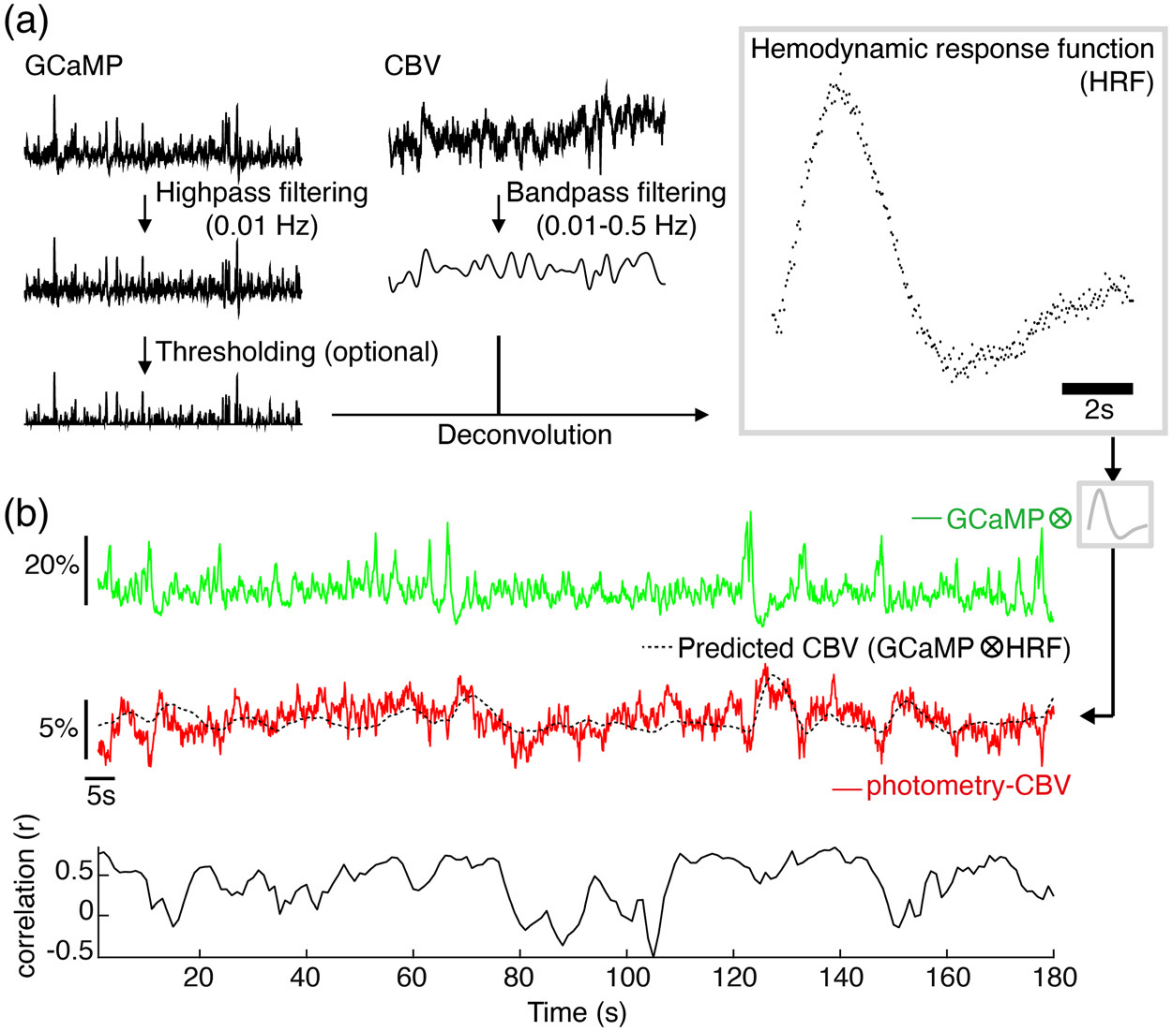
A HRF derived from GCaMP6f and Rhodamine B signals in rat S1 has good predictability for S1 photometry-CBV changes. (a) The pipeline to calculate HRFs from simultaneously recorded GCaMP6f (green) and photometry-CBV (red) time-course. (b) An example of predicted-CBV activity (black) calculated by convolving the derived HRF [shown in (a)] with an independent GCaMP6f time-course (green), the predicted-CBV activity shows a high degree of correlation over time (correlation sliding window width = 5 s) with the corresponding, independently measured, photometry-CBV activity (red). Note that sometimes we observed flipping correlations between positive and negative. Specifically, this instability happened when there was relatively weak neuronal activity within the sliding window, where the CBV changes could be so subtle and buried under random noises. Therefore, the sliding window correlations during weak neuronal activity could be randomly positive or negative.

**Fig. 3:**
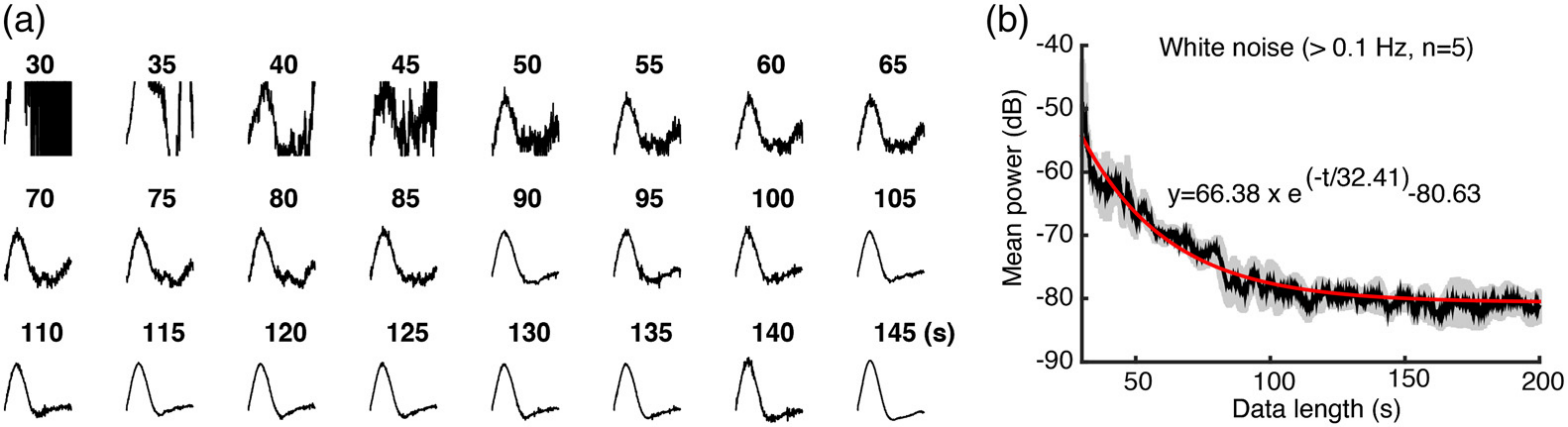
Optimization of the data length of GCaMP6f and photometry-CBV time-courses for calculating HRFs. (a) The resulting HRFs upon regression with different length (s) of GCaMP and CBV time-course. (b) High-frequency white noise (>0.1  Hz) is reduced to a steady-state when the training data are longer than ∼160  s (5 times the decay time constant). We defined the signals >0.1  Hz as noise because hemodynamic activity is commonly considered to be between 0.01-0.1 Hz. Optimization of the data length of GCaMP6f and photometry-CBV time-courses for calculating HRFs (Video 2, MP4, 6 MB [URL: https://doi.org/10.1117/1.NPh.9.3.032205.2]).

Having established our fiber-photometry platform for multimodal recording of neuronal activity and CBV, we sought to determine whether these signals could be readily used to calculate accurate HRFs from resting-state data in the absence of external stimuli. Because we can directly measure spontaneous neuronal activity via the GCaMP6f signal, no stimulation or other timestamped external events should be required to compute HRFs. Figure 2(a) shows the pipeline that we used to derive a HRF from spontaneous GCaMP6f and photometry-CBV signals recorded in S1. The GCaMP6f signal was detrended by high-pass filtering 0.01 Hz, and the photometry-CBV signal was band-pass filtered between 0.01 and 1 Hz to remove low-frequency drift and high-frequency physiological noise. Next, we selected an independent GcAMP6f and photometry-CBV dataset from S1, then calculated the predicted CBV response (black trace) by convolving the independent GCaMP6f trace (green trace) with the HRF model derived using the steps in Fig. 2(a). Importantly, there was a high degree of agreement between the measured photometry-CBV (red trace) and the predicted CBV resting-state signals, confirming the accuracy of the calculated HRF [Fig. 2(b)]. To further optimize our platform and demonstrate the robustness of our pipeline, we empirically determined the resting-state data length required to derive a stable HRF. We used different lengths of simultaneously recorded GCaMP6f and photometry-CBV time-courses to calculate HRFs and their respective noise levels, and found that HRFs are most stable when the input data-length is longer than ∼3  min (Fig. 3, Video 2).

**Fig. 4:**
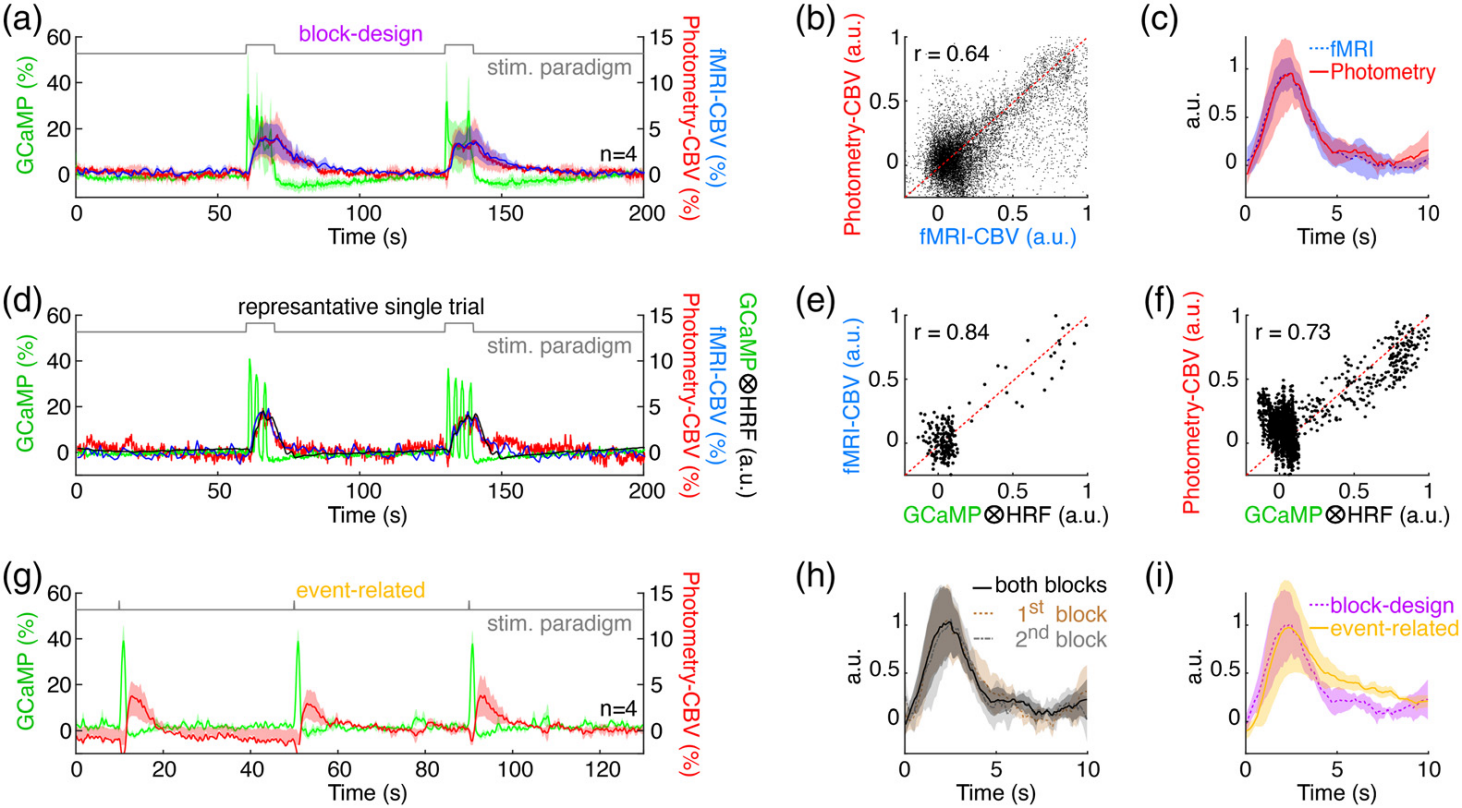
Cross-validation of HRFs derived from photometry-CBV and fMRI-CBV signal changes, or using different stimulation paradigms (n=4). (a) Simultaneously measured fMRI-CBV (blue), photometry-CBV (red) and GCaMP6f (green) time-courses from rat S1, aligned to block-design electrical forepaw stimulation paradigm (gray). (b) Photometry-CBV and fMRI-CBV signals from S1 during blocks of electrical forepaw stimulation are highly correlated (CBV time-courses from the four rats, two repetitions, are all normalized to individual maximum then pooled together). (c) Excellent agreement (ICC=0.99) was identified between the HRFs derived using fMRI-CBV (blue) and photometry-CBV (red). (d) Representative simultaneously measured fMRI-CBV (blue), photometry-CBV (red), and GCaMP6f (green) time-courses from rat S1, aligned to blocks of electrical forepaw stimulation. It should be noted that GCaMP signal drop below baseline after stimulation, likely due to hemoglobin absorption.^27^^,^^28^^,^^43^ The predicted-CBV time-course (black) calculated by convolving the GCaMP6f time-course with the photometry-CBV HRF (c), red is also shown. (e) The predicted-CBV via GCaMP6f time-course and the photometry-CBV HRF has a high correlation with the corresponding photometry-CBV time-course from the same independent dataset. (f) The predicted-CBV time-course calculated by convolving the GCaMP6f time-course with the fMRI-CBV HRF (c), blue also has a high correlation with the corresponding fMRI-CBV time-course from the same independent dataset. The CBV fluctuation around zero, might be due to spontaneous hemodynamic fluctuations by non-neural processes as described in the previous study.^60^ (g) Simultaneously measured fMRI-CBV (blue), photometry-CBV (red) and GCaMP6f (green) time-courses from rat S1, aligned to event-related forepaw stimulation paradigm (gray). (h) Excellent agreement (ICC=0.95) was identified between the HRFs derived using the photometry signals recorded during both blocks (0 to 200 s), first block (31-100 s), and second block (101-170 s) in (a). (i) Good agreement (ICC=0.82) was identified between the HRFs derived using the photometry signals of block-design (a) and event-related (g) stimulation paradigm. In all figures, the shaded area represents standard error. ICC agreement guideline given by Koo and Li (2016):^61^ below 0.50 = poor, between 0.50 and 0.75 = moderate, between 0.75 and 0.90 = good, above 0.90 = excellent.

Next, we compared rat S1 HRFs derived from photometry-CBV and fMRI-CBV signal changes aligned to electrical forepaw stimulation to cross-validate the two approaches, thereby highlighting the potential utility of photometry-derived HRFs for fMRI applications [Figs. 4(a)–4(c)]. Because the fMRI sampling rate was 1 Hz, we interpolated the fMRI-CBV time-courses to 10 Hz to match the temporal resolution of the GCaMP6f and photometry-CBV signals. As expected, photometry-CBV and fMRI-CBV signals were also significantly correlated over time [Fig. 4(b)]. Most importantly, we found minimal differences between HRFs derived from photometry-CBV and fMRI-CBV [ICC=0.99, Fig. 4(c)]. Further, convolution of the measured GCaMP6f signal from a representative independent GCaMP6f, photometry-CBV, and fMRI-CBV dataset [Fig. 4(d)] and either the photometry-CBV or fMRI-CBV derived HRF [Fig. 4(c)] both produced a predicted-CBV time-course that was well correlated with the corresponding measured CBV time-course [Figs. 4(e) and 4(f)]. In addition, to understand how much the resolved HRF is influenced by potential neuronal habituation due to repetitive electrical forepaw stimulation, we evaluate the similarity among the HRFs derived using the first, second, and both stimulation blocks [Fig. 4(a)], and found excellent agreement among these three HRFs [ICC=0.95, Fig. 4(h)]. Similarly, the HRFs obtained from block-design [Fig. 4(a)] and event-related [Fig. 4(g)] forepaw stimulation also showed excellent agreement to each other [ICC=0.82, Fig. 4(i)].

**Fig. 5:**
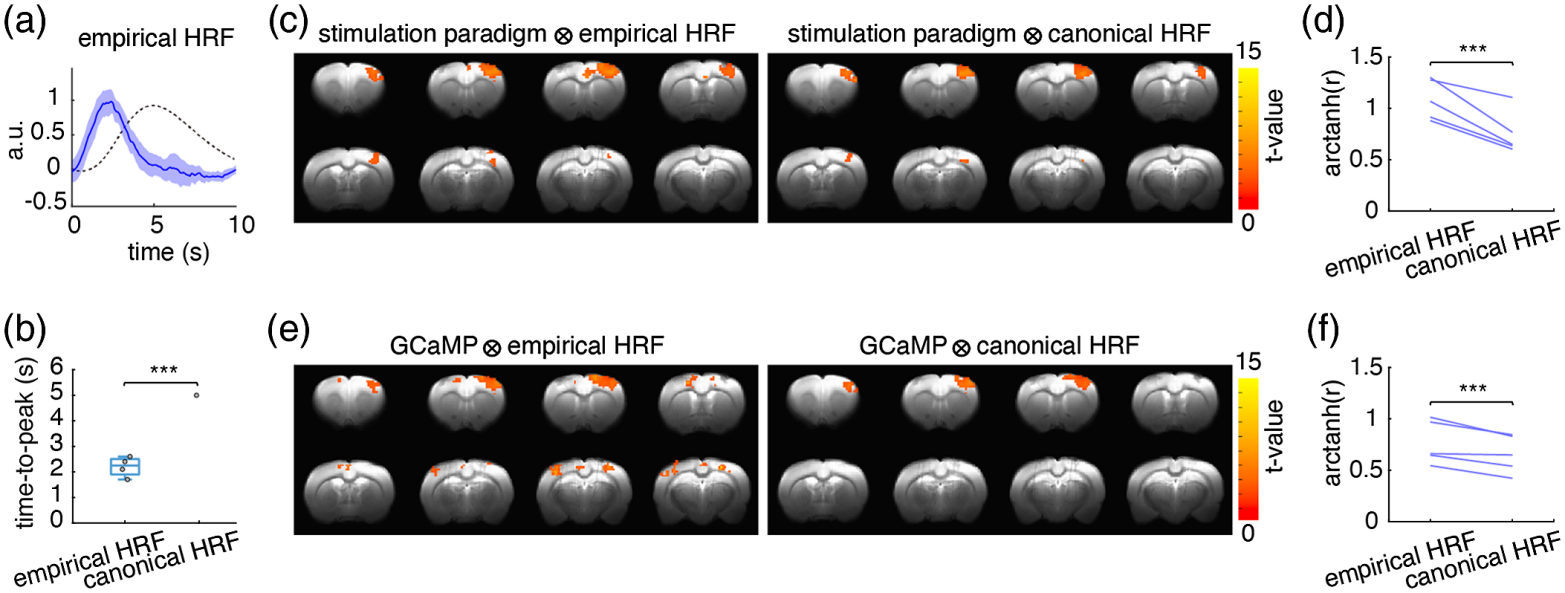
Using the fiber-photometry derived empirical HRF for fMRI analyses improved detection of S1 activation clusters and downstream activity compared to analyses using the canonical HRF. (a) The rat S1 empirical HRF (blue, the shaded area represents standard error) derived using S1 GCaMP and photometry-CBV signals was substantially narrower than the human-based canonical HRF (black dashed line), which is implemented in the SPM package for brain data analysis by default.^11^ (b) The time-to-peak of the empirical HRF was significantly shorter than the canonical HRF (p<0.001). (c) The detected S1 activation cluster size was larger when the stimulation paradigm was convolved with the empirical HRF than with the canonical HRF for GLM analysis of the fMRI data ($p<0.05_{\text{corrected}}$). (d) The correlation between the regressor-generated CBV time-course and the fMRI-CBV time-course measured from the corresponding S1 activation cluster was significantly higher when using the empirical HRF versus the canonical HRF for regressor calculation. The correlations were Fisher transformed to meet the requirement as normal distribution for Student T-test and shown in arctanh(r), p<0.001. (e) A significant activation cluster was detected in bilateral PPC by convolving the GCaMP6f signal with the empirical HRF but not the canonical HRF ($p<0.05_{\text{corrected}}$). (f) The correlation between the regressor-generated CBV time-course and the fMRI-CBV time-course measured from the corresponding S1 activation cluster was significantly higher when using the empirical HRF and GCaMP6f signal versus the canonical HRF and GCaMP6f for regressor calculation The correlations were Fisher transformed to meet the requirement as normal distribution for Student T-test and shown in arctanh(r), p<0.001.

An important metric for the utility of empirical HRFs is their performance relative to the canonical HRF in capturing whole-brain activity changes within fMRI datasets. Notably, the empirical HRF from rat S1 photometry-CBV data in Fig. 4(c) was narrower in appearance than the canonical HRF, with a significantly shorter time to peak [Figs. 5(a)–5(b)]. To understand how these differences between the fiber-photometry derived empirical HRF and the canonical HRF could impact fMRI analyses, we compared their performance in calculating forepaw stimulation-induced brain activation maps. We first convolved the stimulation paradigm to each HRF, then used the products as regressors for GLM analyses of fMRI data. We found that the group-level statistical map for brain activation calculated with the empirical HRF detected a larger activation cluster in S1 [Fig. 5(c) left, 240 voxels] compared to the map derived using the canonical HRF [Fig. 5(c), right, 161 voxels]. Further, there was a higher correlation between the regressor calculated using the empirical HRF than with the canonical HRF and the corresponding S1 fMRI-CBV changes over time [Fig. 5(d)]. Next, we repeated the same analyses, but generated the regressors by convolving the HRFs with GCaMP6f signals instead of the stimulation paradigm. In contrast to the previous analyses, this approach produced one unique regressor for each recorded stimulation trial according to the measured GCaMP6f neuronal activity. In result, we observed significant S1 activation clusters using both HRFs as before, with an additional activation cluster unique to the empirical HRF analysis that was located in the bilateral post parietal cortices (PPC), which receives substantial inputs from the S1 forelimb area^62^ [Fig. 5(e)]. Additionally, similar to the previous results [Figs. 5(c)–5(d)], analysis with empirical HRF detected a larger S1 activation cluster [Fig. 5(e) left, 289 voxels] in the group-level statistical brain activation map compared to the canonical HRF [Fig. 5(e) right, 149 voxels], and the empirical HRF derived regressor also showed higher correlation with the corresponding S1 fMRI-CBV measurements [Fig. 5(f)].

**Fig. 6:**
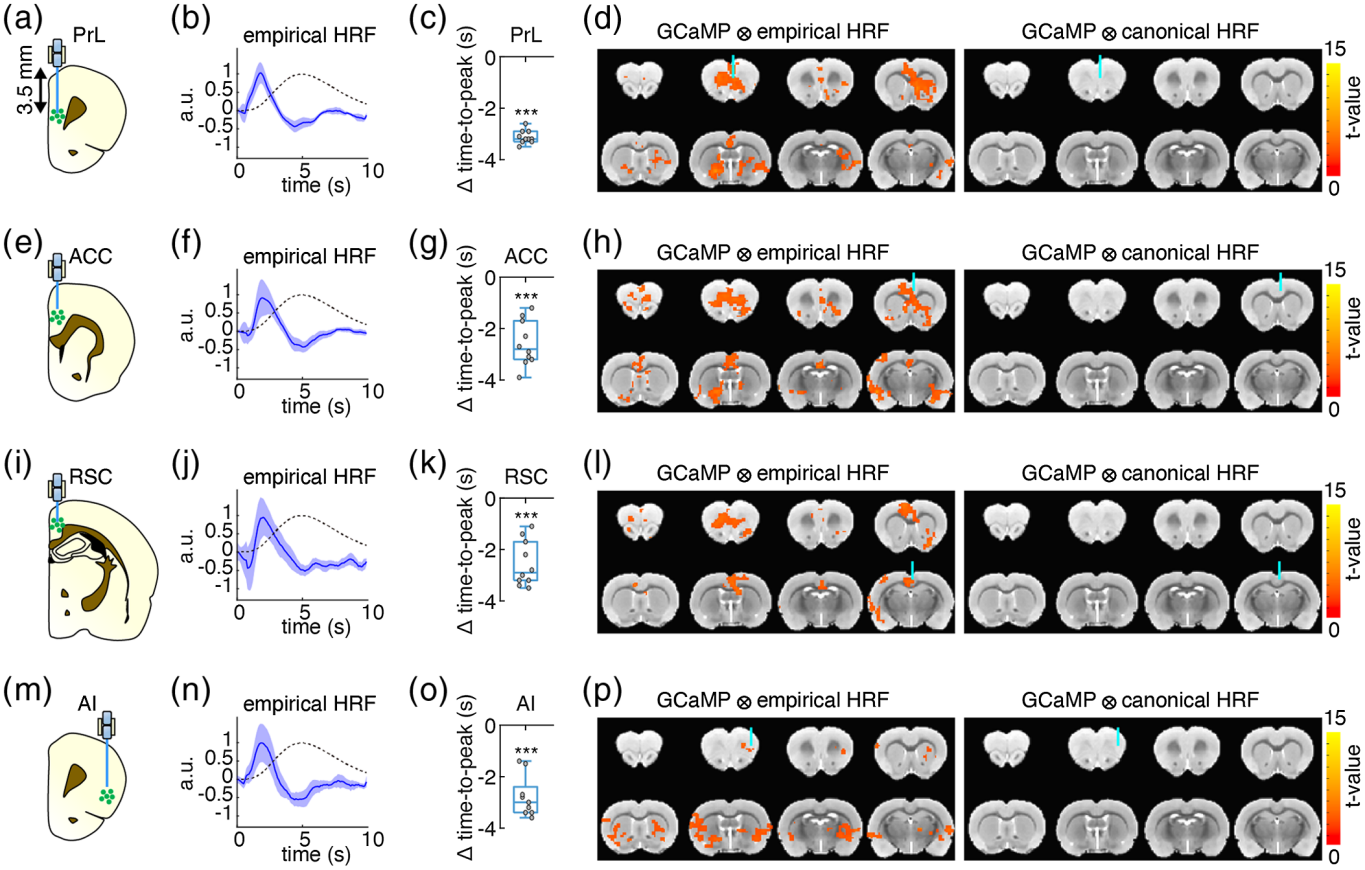
(a, e, i, and m) Fiber-photometry recording sites in rat cortical areas used for calculating empirical HRFs. Each rat was implanted fibers in PrL, ACC, RSC, and AI (n=10, each rat was recorded two repetitions of 10 min resting-state). (b, f, j, n) The empirical HRFs derived resting-state GCaMP6f and Rhodamine B time-course data recorded by fiber-photometry (blue solid line, the shaded area represents standard error) versus the canonical HRF (black dashed line). (c, g, k, o) The time-to-peak differences of the empirical HRFs versus canonical HRF (Δ time-to-peak); negative values indicate time-to-peak latencies shorter than the canonical HRF (all p<0.001). (d, h, l, p) GLM analyses of fMRI data using spontaneous GCaMP6f signals convolved with the empirical HRFs as regressors detected brain-wide functional networks (left panels), which could not be detected using the same GCaMP6f signals convolved with the canonical HRF as regressors (right panels) ($p<0.05_{\text{corrected}}$, n=7). Note that the GLM coefficients below each optic fiber targeting site were not the strongest among the whole functional networks (left panels). It is likely due to confounds associated with fiber implantation and/or susceptibility effect cause by fiber, which is commonly observed in fMRI studies with optic fiber implant.^44^^–^^46^

One unique advantage of fiber-photometry over other optical techniques is that HRFs can be probed at deep brain regions because there is no tissue-depth limitation to the signals received. To illustrate this application, we provide an example of a HRF derived from spontaneous GCaMP6f and photometry-CBV signals in the PrL, where the targeted site was 3.5-mm deep from brain surface in rats [Figs. 6(a) and 6(b)]. By convolving the empirical photometry-derived HRF with the PrL GCaMP signal to use as a regressor in GLM analysis of simultaneously acquired fMRI data, we were able to extract brain regions related to the default mode network (DMN), such as RSC, as well as regions functionally connected to the PrL [Fig. 6(d), left]. However, when using the canonical HRF for regressor calculation, the DMN-like pattern failed to be extracted via the same process [Fig. 6(d), right].

**Fig. 7:**
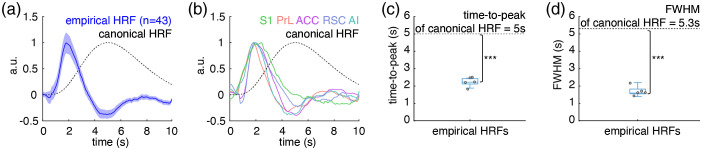
The average empirical rat cortical HRF derived from fiber-photometry GCaMP6f and Rhodamine B signal in rat S1, PrL, ACC, RSC, and AI is significantly faster and narrower than the canonical HRF. (a) The average empirical rat cortical HRF (blue solid line, the shaded area represents standard error) obtained from 44 total photometry recordings versus the canonical HRF (black dash line). The average empirical rat cortical HRF was pooled from HRFs in S1: n=4/2 repetitions, PrL/ACC/RSC/AI: n=10/2 repetitions. In addition to the S1 HRF, the HRFs of PrL, ACC, RSC, and AI were computed from the same group of animals using a four-channel recording system. The intraindividual HRFs were first averaged, then these average individual HRFs were used to calculate the average empirical rat cortical HRF. (b) The difference in shape between the empirical HRFs for individual cortical areas (colored solid lines) appears to be substantially smaller than between each HRF and the canonical HRF (black dashed line). (c) The time-to-peak latencies of the empirical HRFs are significantly shorter than the canonical HRF (p<0.001). (d) The FWHMs of the empirical HRFs are significantly narrower than the canonical HRF (p<0.001).

As it has been postulated that HRFs can be region-specific, we repeated the previous experiment with fiber-photometry recordings from three more cortical areas in the rat brain, including: the ACC, the RSC, and the AI. For each, the empirical HRF performed better in the fMRI GLM analysis than the canonical HRF, with DMN-like patterns from the ACC and RSC HRFs [Figs. 6(e)–6(l)], we found a distinct pattern from the AI HRF [Figs. 6(m)–6(p)], and no pattern from the canonical HRF [Figs. 6(e)–6(p)]. Similar to the empirical HRF derived from S1 photometry data displayed in Fig. 5(a), the empirical HRFs for the four additional cortical areas reported in Fig. 6 also exhibited a significantly shorter time-to-peak latency than the canonical HRF [Figs. 6(b) and 6(c), 6(f) and 6(g), 6(j) and 6(k), and 6(n) and 6(o)]. Specifically, in contrast to the typical 5-s time-to-peak latency of the canonical HRF,^63^^,^^64^ the time-to-peak latencies for the empirical HRFs were as follows: S1=2.2±0.23  s, PrL=1.88±0.09  s, ACC=2.42±0.29  s, RSC=2.45±0.29  s, and AI=2.22±0.29  s. Intriguingly, among these empirically measured HRFs from rat cortical regions, no significant difference was identified using the functional t-test (a statistical test developed for comparing HRFs,^19^ which uses a pointwise test approach based on a permutation method) (p=0.66). We further evaluated the similarity among these empirical HRFs using ICC^61^ and found excellent agreement (ICC=0.95) among these empirical HRFs. Given the similarity between empirical HRFs for rat cortical regions, we pooled these five HRFs together to generate an average rat cortical HRF (Table 1) for straightforward dissemination. The average rat cortical HRF had a time-to-peak latency of 2.23±0.11  s, and a FWHM of 1.7±0.15  s, which is significantly faster and narrower than the canonical HRF with a 5 s time-to-peak and a 5.3 s FWHM, respectively (Fig. 7).

**Table 1:** The average empirical rat cortical HRF derived from fiber-photometry GCaMP6f and Rhodamine B signal in rat S1, PrL, ACC, RSC, and AI.

Time (s)	Empirical HRF
0	0.000
0.1	−0.001
0.2	−0.012
0.3	−0.026
0.4	−0.039
0.5	−0.045
0.6	−0.023
0.7	0.050
0.8	0.069
0.9	0.117
1	0.184
1.1	0.250
1.2	0.356
1.3	0.485
1.4	0.624
1.5	0.761
1.6	0.870
1.7	0.948
1.8	0.995
1.9	1.000
2	0.979
2.1	0.940
2.2	0.889
2.3	0.844
2.4	0.792
2.5	0.739
2.6	0.658
2.7	0.588
2.8	0.524
2.9	0.438
3	0.363
3.1	0.284
3.2	0.221
3.3	0.150
3.4	0.098
3.5	0.042
3.6	−0.009
3.7	−0.048
3.8	-0.088
3.9	−0.140
4	−0.182
4.1	−0.232
4.2	−0.268
4.3	−0.294
4.4	−0.322
4.5	−0.323
4.6	−0.335
4.7	−0.338
4.8	−0.340
4.9	−0.345
5	−0.343
5.1	−0.339
5.2	−0.340
5.3	−0.330
5.4	−0.313
5.5	−0.294
5.6	−0.276
5.7	−0.252
5.8	−0.225
5.9	−0.210
6	−0.184
6.1	−0.167
6.2	−0.151
6.3	−0.139
6.4	−0.122
6.5	−0.120
6.6	−0.115
6.7	−0.112
6.8	−0.110
6.9	−0.102
7	−0.103
7.1	−0.097
7.2	−0.099
7.3	−0.083
7.4	−0.074
7.5	−0.069
7.6	−0.056
7.7	−0.054
7.8	−0.048
7.9	−0.024
8	0.000

## 4 Discussion

fMRI studies in rodents provide a unique opportunity to selectively interrogate and monitor large-scale activity changes, which is otherwise challenging to accomplish in humans.^65^ While emerging evidence shows that neurovascular coupling can be substantially different across species, brain regions, and neurophysiological conditions,^5^^,^^6^ a canonical human HRF is still used in a majority of rodent studies. Current attempts to calculate empirical HRFs from multi-modal measurement-based approaches are limited either by fMRI-compatibility issues (e.g., electrophysiology^20^^–^^25^), or by signal loss through brain tissue (e.g., multi-photon microscopy^26^^,^^29^^–^^33^ and wide-field optical imaging^27^^,^^28^), whereas fMRI data-driven approaches are heavily influenced by model selection.^15^^–^^19^ In this study, we addressed these limitations by implementing a fMRI-compatible spectral fiber-photometry platform, which allows for simultaneous optical ground-truth measurement of neuronal activity and CBV changes from any target in the brain. Our results demonstrated that data from this platform can be used to calculate empirical HRFs with a standard pipeline and with sufficient efficiency to use resting-state datasets without discrete stimulation events [Figs. 2, 3, 6, S1 (Video 1), and Video 2]. Further, we revealed that empirical HRFs derived from our platform consistently outperform the canonical HRF in GLM analyses of simultaneously acquired rat brain fMRI data.

**Fig. 8:**
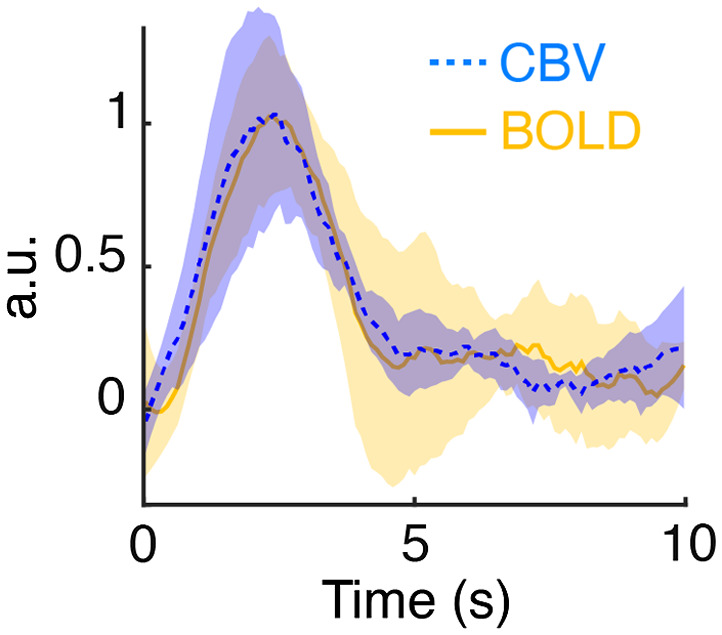
The CBV-based and BOLD-based HRFs, derived using block-design forepaw stimulation data, showed excellent agreement with each other (ICC=0.95, n=4).

Our results indicate that while empirical HRFs from rat cortical areas, including the S1, PrL, ACC, RSC, and AI are generally similar. Nevertheless, it does not necessarily suggest identical HRF should be applied across all the nuclei in the rodent brain, as shown in one of our recent studies.^66^ Among the cortical HRF measured in this study, we found their time-to-peak latencies and FWHMs are significantly shorter than the widely used canonical HRF,^11^ which was derived based on the human studies by Friston and colleagues.^63^^,^^64^ Indeed, the peak and FWHM of our cortical empirical HRFs are consistent with other previously reported, empirical HRFs identified for rat S1,^67^^,^^68^ visual cortex,^69^ and olfactory bulb.^34^ Previous studies have suggested that the faster dynamics of rat cortical HRFs compared to the human-derived canonical HRF may be due to a higher CBF velocity in rodent capillary and venous structures, as well as a relatively larger contribution of large vessel effects to the signal.^67^^,^^68^ It is important to note that the current study utilized CBV-fMRI to match with photometry CBV recording, and a kinetic difference may exist between the canonical BOLD-derived HRF.^63^^,^^64^ Nevertheless, a recent study by Peng et al.^12^ demonstrated that fMRI detection performance between CBV-based or BOLD-based HRFs are not significantly different. It has also been reported that differences in time-to-peak estimate might have to be on the order of 2.5 s to cause false negative findings.^7^ Furthermore, studies comparing empirical BOLD and CBV HRFs have only reported relatively small differences in shape and timing compared to those observed here.^67^^,^^70^^,^^71^ Indeed, our results from a subset of experiments using BOLD measures are in agreement with these findings and show minimal discrepancies between BOLD- and CBV-derived HRF (Fig. 8). Notably, we identified the time-to-peak of our empirical HRFs to be ∼2.8  s earlier than that of the canonical HRF (Fig. 7). This misprediction will cause dramatically lower detection performance of fMRI changes by GLM analyses using canonical rather than empirical HRF-derived regressors, as demonstrated by the relatively smaller S1 activation cluster sizes from electrical forepaw stimulation or related GCaMP time-courses in Fig. 5 and complete absence of detected features from resting-state data in Fig. 6. Similarly, use of inaccurate HRFs may also substantially impact DCM^72^ and granger causality^73^ results in fMRI data analysis.

**Fig. 9:**
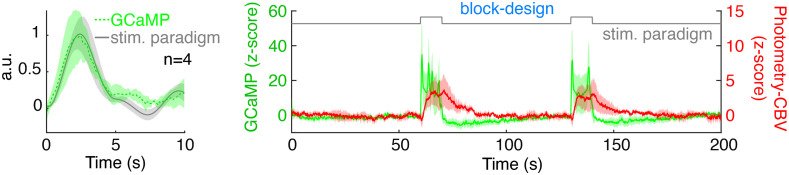
Excellent agreement (ICC=0.94) was identified between the HRFs derived using GCaMP6f (green) and stimulation paradigm (gray).

Our empirical HRFs are generally consistent with previously reported rat HRFs.^34^^,^^67^^–^^69^ However, it should be noted that the HRFs presented herein represent vascular transfer functions of GCaMP6f activity rather than canonical impulse-response functions where the ”impulse” is supposed to be brief and nearly instantaneous (e.g., spiking or neurons). As GCaMP inherently has delayed response kinetics relative to neuronal spiking activity,^14^ one could assume this offset may contributes to the shorter time-to-peak latency of our empirical HRFs compared to the canonical HRF as shown in Fig. 7. Nevertheless, in vivo evidence using GCaMP6f, the variant employed in this study, shows the time-to-peak to be only 40 ms following neuronal spiking events,^14^ whereas the time-to-peak difference between the average of our empirical HRFs and the canonical HRF was ∼2.8  s (Fig. 7). Therefore, the contribution of GCaMP6f kinetics and calcium dynamics to our empirical HRFs, while present, is thought to be insignificant to the hemodynamic time-scale. Indeed, the HRF derived from evoked GCaMP6f activity and stimulation paradigm showed minimal differences between each other (Fig. 9).

As the proposed empirical HRFs are derived using GCaMP6f signals, it is expected that regressors generated by convolving GCaMP6f signals with their corresponding empirical HRFs would perform better than regressors generated by convolving GCaMP6f signals and the canonical HRF. Indeed, in Fig. 5, we demonstrate that out of the possible combinations of either the GCaMP6f signals or the stimulation paradigm and the S1 empirical HRF or the canonical HRF for regressor calculation, only the GCaMP6f and empirical HRF combination captured PPC activation in the simultaneously acquired fMRI data. Given that the PPC receives substantial inputs from the S1 forelimb area,^62^ and GCaMP6f is thought to measure neuronal output activity,^74^ GCaMP6f and empirical HRF-derived regressors may prove particularly useful for reliable extraction of downstream activity changes from targeted efferent neuron populations. Nevertheless, our results also suggest better detection performance in fMRI data when using the stimulation paradigm and S1 empirical HRF rather than the canonical HRF in regressor calculation. Taken together, these findings suggest that empirical HRFs derived from concurrently measured neuronal activity and CBV changes may be more accurate for analyzing rat fMRI data than using the human-based canonical HRF.

**Fig. 10:**
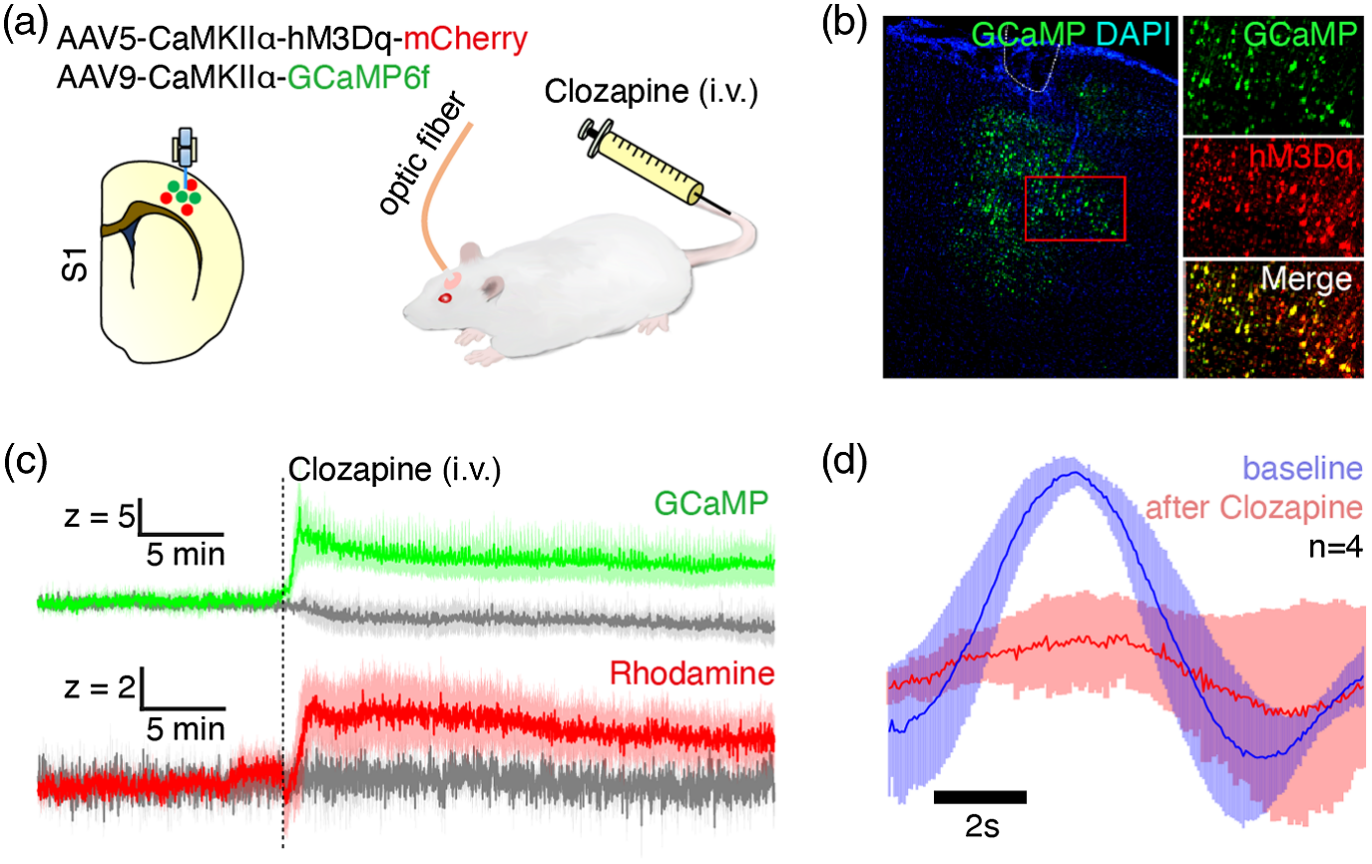
Our technique provides an opportunity to address the hypothesis that HRFs are state dependent. Here, we used AAV vectors to co-express hM3Dq and GCaMP6f in rat S1 under the CaMKIIα promotor. (a) GcAMP6f combined with Rhodamine B injection enabled simultaneous measurement of neuronal activity and photometry-CBV, respectively, while hM3Dq activation by clozapine (0.05  mg/kg, i.v.) allowed the induction of an up-regulated state of local neuronal activity. (b) Histological evidence of colocalized expression of GCaMP6f and hM3Dq in S1 principal neurons. (c) Clozapine enhanced S1 GCaMP6f signal and caused a robust increase in S1 photometry-CBV. Note that clozapine is also an antipsychotic drug, yet used here to activate the hM3Dq receptors.^89^ (d) Importantly, an empirical HRF calculated from spontaneous resting-state activity time-courses from S1 after clozapine injection was significantly attenuated compared to an empirical HRF calculated from time-courses before clozapine injection, suggesting that HRFs can be state dependent.

Previous studies have shown that HRFs can vary across brain regions in both humans^7^ and rodents.^8^ While we did not identify any significant differences in empirical HRFs across five cortical regions in the rat brain (i.e., S1, PrL, ACC, RSC, and AI), future investigations of subcortical regions, which can be readily probed with our platform, may yield divergent results. For example, negative, rather than positive vascular responses to increased neuronal activity have been reported in the hippocampus,^75^ caudate-putamen,^6^^,^^43^^,^^76^^,^^77^ and different hemodynamic properties between cortical and subcortical brain regions have also been reported in the rat visual system.^78^ However, it should be noted that the aforementioned studies utilized altered physiological states or external stimulations to characterize the regional neurovascular relationships, and these relationships may be different under resting-state conditions. In support, altered vascular responses have been observed in several disease states,^77^^,^^79^^,^^80^ and in response to stress,^81^ pain,^8^ or anesthesia,^82^ and many studies have also shown non-linear coupling between sensory stimulation frequencies and hemodynamic responses.^5^^,^^69^^,^^83^^–^^87^ Furthermore, there are known interactions between neurophysiological conditions and neuronal responses to sensory stimulations.^9^^,^^10^ Namely, neural adaptations during prolonged stimulations can vary according to the stimulation frequencies and the anesthetics being used.^84^^,^^88^ Our fiber-photometry platform is well-suited for clarifying HRF differences across brain regions and conditions. With the option to efficiently derive empirical HRFs from resting-state data, potential confounds related to sensory or external stimulation and physiological state-changes can be avoided with the use of our platform. Alternatively, because our platform simultaneously measures local neuronal activity and CBV changes, alterations in stimulation-responses or neurophysiological state can be detected for removal or further examination in HRF calculations (Fig. 10). In addition, our platform allows computing empirical HRF in awake, freely moving rodents. This might be particularly useful in awake-rodent-fMRI studies, where the HRF is likely to differ from anesthetized condition^82^ and stimulation-based HRF calibration might be unfeasible.

The fiber-photometry platform characterized in this study allows for the simultaneous collection of GCaMP6f green-fluorescent neuronal activity signals and Rhodamine B red-fluorescent CBV change signals from a single implanted optical fiber. Traditional fiber-photometry platforms rely on single-point fluorescence intensity measurements via photodiodes and do not provide sufficient information to fully separate independent signals with partially overlapping spectra. Therefore, to record neuronal activity and CBV changes with the accuracy needed for HRF calculations, our platform instead uses a spectrometer recording device to capture the entire fluorescence emission spectrum, allowing us to separate distinct signals via linear-unmixing. Importantly, while we focus primarily on calculating empirical HRFs from GCaMP and photometry-CBV, it is worth mentioning that this approach can be adapted and scaled to concurrently measure signal with distinct spectra from a variety of fluorescent sources. As such, future investigations with our platform could leverage the rapidly expanding library of genetically encoded neuronal activity and/or neurochemical sensors to shed important light on how selected activity modulates vascular tone. To this end, the deconvolution pipeline proposed herein is capable of calculating HRF using other fluorescent sensor activities such as dopamine,^90^ norepinephrine,^91^ glutamate,^92^^,^^93^ GABA,^94^ etc. Additionally, our platform could also be used to compute transfer functions between any combination of sensors with distinct spectra (e.g., between neuronal activity and neurochemical release). Several red-shifted fluorescent sensors such as jRGECO^95^ and iGECI^96^^,^^97^ would be ideal for such applications.

## 5 Conclusion

We have established an fMRI-compatible, spectral, fiber-photometry platform for HRF calculation and validation. We show that empirical HRFs derived from neuronal activity and CBV changes recorded from rat cortical areas have significantly faster kinetics than the widely used canonical human HRF, and demonstrate superior detection performance of these empirical HRFs over the canonical HRF in GLM analyses of rat fMRI data. HRF calculations with this platform can be conducted from resting-state or stimulation-based recording conditions and for any target in the brain. This platform is also readily scalable to multiple simultaneous recording sites and adaptable for the study of transfer functions between stimulation events, neuronal activity, neurotransmitter release, and hemodynamic responses. Through this work, we make our fiber-photometry platform design and data analysis pipeline publicly available, with the hope that this information will facilitate the advancement of fiber-photometry and HRF-calculation methods and the adoption of empirical HRFs for use in future animal fMRI studies.

## Code, data, and Materials Availability

The codes used in this study are available on GitHub: https://github.com/CAMRIatUNC/Photometry-tools. The data that support the findings of this study are available from the authors upon reasonable request.
